# First release of the European marine omics biodiversity observation network (EMO BON) shotgun metagenomics data from water and sediment samples

**DOI:** 10.3897/BDJ.13.e143585

**Published:** 2025-03-12

**Authors:** Christina Pavloudi, Ioulia Santi, Iñigo Azua, Zuriñe Baña, Mauro Bastianini, Caroline Belser, Jone Bilbao, Julie Bitz-Thorsen, Caroline Broudin, Mathieu Camusat, Ibon Cancio, Louis Caray-Counil, Raffaella Casotti, Jade Castel, Thierry Comtet, Cymon J Cox, Claire Daguin, Oihane Díaz de Cerio, Katrina Exter, Cécile Fauvelot, Miguel J. Frada, Pierre E Galand, Laurence Garczarek, Jose González Fernández, Laure Guillou, Pascal I. Hablützel, Hanneloor Heynderickx, Céline Houbin, Anne Emmanuelle Kervella, Apostolos Krystallas, Rune Lagaisse, Arnaud Laroquette, Lyvia Lescure, Eva Lopes, Melina Loulakaki, Bruno Louro, Catarina Magalhaes, Maria Maidanou, Francesca Margiotta, Marina Montresor, Fabrice Not, Estefanía Paredes, Isabella Percopo, Erwan Péru, Julie Poulain, Kim Præbel, Fabienne Rigaut-Jalabert, Sarah Romac, Melanthia Stavroulaki, Jesús Souza Troncoso, Eric Thiébaut, Wilfried Thomas, Andrzej Tkacz, Anna Chiara Trano, Patrick Wincker, Nicolas Pade

**Affiliations:** 1 European Marine Biological Resource Centre (EMBRC-ERIC), Paris, France European Marine Biological Resource Centre (EMBRC-ERIC) Paris France; 2 Institute of Marine Biology, Biotechnology and Aquaculture (IMBBC), Hellenic Centre for Marine Research (HCMR), Heraklion, Greece Institute of Marine Biology, Biotechnology and Aquaculture (IMBBC), Hellenic Centre for Marine Research (HCMR) Heraklion Greece; 3 Department of Immunology, Microbiology and Parasitology, Faculty of Science and Technology and Research Centre for Experimental Marine Biology and Biotechnology, Plentziako itsas Estazioa (PiE-UPV/EHU), University of the Basque Country (UPV/EHU), Biscay, Spain Department of Immunology, Microbiology and Parasitology, Faculty of Science and Technology and Research Centre for Experimental Marine Biology and Biotechnology, Plentziako itsas Estazioa (PiE-UPV/EHU), University of the Basque Country (UPV/EHU) Biscay Spain; 4 CNR-National Research Council, ISMAR - Institute of Marine Sciences, Venice, Italy CNR-National Research Council, ISMAR - Institute of Marine Sciences Venice Italy; 5 Génomique Métabolique, Genoscope, Institut François Jacob, Commissariat à l’Energie Atomique (CEA), CNRS, Université Evry, Université Paris-Saclay, 2 Rue Gaston Crémieux, 91057, Evry, France Génomique Métabolique, Genoscope, Institut François Jacob, Commissariat à l’Energie Atomique (CEA), CNRS, Université Evry, Université Paris-Saclay, 2 Rue Gaston Crémieux, 91057 Evry France; 6 Department of Plant Biology and Ecology, Faculty of Science and Technology and Research Centre for Experimental Marine Biology and Biotechnology, Plentziako itsas Estazioa (PiE-UPV/EHU), University of the Basque Country (UPV/EHU), Biscay, Spain Department of Plant Biology and Ecology, Faculty of Science and Technology and Research Centre for Experimental Marine Biology and Biotechnology, Plentziako itsas Estazioa (PiE-UPV/EHU), University of the Basque Country (UPV/EHU) Biscay Spain; 7 Norwegian College of Fishery Science, UiT The Arctic University of Norway, Tromsø, Norway Norwegian College of Fishery Science, UiT The Arctic University of Norway Tromsø Norway; 8 Sorbonne Université, CNRS, FR2424, Station Biologique de Roscoff, 29680, Roscoff, France Sorbonne Université, CNRS, FR2424, Station Biologique de Roscoff, 29680 Roscoff France; 9 Department of Zoology and Animal Cell Biology, Faculty of Science and Technology and Research Centre for Experimental Marine Biology and Biotechnology, Plentziako itsas Estazioa (PiE-UPV/EHU), University of the Basque Country (UPV/EHU), Biscay, Spain Department of Zoology and Animal Cell Biology, Faculty of Science and Technology and Research Centre for Experimental Marine Biology and Biotechnology, Plentziako itsas Estazioa (PiE-UPV/EHU), University of the Basque Country (UPV/EHU) Biscay Spain; 10 Sorbonne University, CNRS, Laboratoire d’Océanographie de Villefranche, Villefranche-sur-Mer, France Sorbonne University, CNRS, Laboratoire d’Océanographie de Villefranche Villefranche-sur-Mer France; 11 Integrative Marine Ecology Department, Stazione Zoologica Anton Dorn, Naples, Italy Integrative Marine Ecology Department, Stazione Zoologica Anton Dorn Naples Italy; 12 Sorbonne Université, CNRS, Adaptation et Diversité en Milieu Marin, AD2M, 29680, Roscoff, France Sorbonne Université, CNRS, Adaptation et Diversité en Milieu Marin, AD2M, 29680 Roscoff France; 13 Centro de Ciências do Mar, Universidade do Algarve, Faro, Portugal Centro de Ciências do Mar, Universidade do Algarve Faro Portugal; 14 Flanders Marine Institute [Vlaams Instituut voor de Zee (VLIZ)], Ostend, Belgium Flanders Marine Institute [Vlaams Instituut voor de Zee (VLIZ)] Ostend Belgium; 15 UMR ENTROPIE (IRD, Université de La Réunion, CNRS, IFREMER, Université de la Nouvelle-Calédonie), Villefranche-sur-Mer, France and Sorbonne University, CNRS, Laboratoire d’Océanographie de Villefranche, Villefranche-sur-Mer, France UMR ENTROPIE (IRD, Université de La Réunion, CNRS, IFREMER, Université de la Nouvelle-Calédonie), Villefranche-sur-Mer, France and Sorbonne University, CNRS, Laboratoire d’Océanographie de Villefranche Villefranche-sur-Mer France; 16 Department of Ecology, Evolution and Behavior, Silberman Institute of Life Sciences, The Hebrew University of Jerusalem, Jerusalem, Israel and The Interuniversity Institute for Marine Sciences in Eilat, Eilat, Israel Department of Ecology, Evolution and Behavior, Silberman Institute of Life Sciences, The Hebrew University of Jerusalem, Jerusalem, Israel and The Interuniversity Institute for Marine Sciences in Eilat Eilat Israel; 17 Sorbonne Université, CNRS, Laboratoire d’Ecogéochimie des Environnements Benthiques (LECOB), Observatoire Océanologique de Banyuls, Banyuls sur Mer, France Sorbonne Université, CNRS, Laboratoire d’Ecogéochimie des Environnements Benthiques (LECOB), Observatoire Océanologique de Banyuls Banyuls sur Mer France; 18 Centro de Investigación Mariña, Universidade de Vigo, Estación de Ciencias Mariñas de Toralla, Vigo, Spain Centro de Investigación Mariña, Universidade de Vigo, Estación de Ciencias Mariñas de Toralla Vigo Spain; 19 Vrije Universiteit Brussel, Brussel, Belgium Vrije Universiteit Brussel Brussel Belgium; 20 CIIMAR–Interdisciplinary Centre of Marine and Environmental Research, University of Porto, Matosinhos, Portugal, Department of Biology, FCUP–Faculty of Sciences of the University of Porto, Porto, Portugal CIIMAR–Interdisciplinary Centre of Marine and Environmental Research, University of Porto, Matosinhos, Portugal, Department of Biology, FCUP–Faculty of Sciences of the University of Porto Porto Portugal; 21 Stazione Zoologica Anton Dohrn, Naples, Italy Stazione Zoologica Anton Dohrn Naples Italy; 22 Department of Ecology and Animal Biology, ECOCOST group, Marine Sciences Faculty, University of Vigo, Vigo, Spain Department of Ecology and Animal Biology, ECOCOST group, Marine Sciences Faculty, University of Vigo Vigo Spain; 23 Sorbonne Université, CNRS, OSU STAMAR, Station Biologique de Roscoff, 29680, Roscoff, France Sorbonne Université, CNRS, OSU STAMAR, Station Biologique de Roscoff, 29680 Roscoff France

## Abstract

The European Marine Omics Biodiversity Observation Network (EMO BON) is an initiative of the European Marine Biological Resource Centre (EMBRC) to establish a persistent genomic observatory amongst designated European coastal marine sites, sharing the same protocols for sampling and data curation. Environmental samples are collected from the water column and, at some sites, soft sediments and hard substrates (Autonomous Reef Monitoring Structures - ARMS), together with a set of mandatory and discretionary metadata (including Essential Ocean Variables - EOVs). Samples are collected following standardised protocols at regular and specified intervals and sequenced in large six-monthly batches at a centralised sequencing facility. The use of standard operating procedures (SOPs) during data collection, library preparation and sequencing aims to provide uniformity amongst the data collected from the sites. Coupled with strict adherence to open and FAIR (Findable, Accessible, Interoperable, Reusable) data principles, this ensures maximum comparability amongst samples and enhances reusability and interoperability of the data with other data sources. The observatory network was launched in June 2021, when the first sampling campaign took place.

## Introduction

Here we report the first data release from the European Marine Omics Biodiversity Observation Network (EMO BON) (Santi et al. 2023). This release includes data derived from water and sediment samples that were collected between June and September 2021 from the 13 observatories, across European seas and the Red Sea.

### Value of the dataset

This dataset includes raw DNA sequences obtained from shotgun metagenomics sequencing of water and sediment samples from 13 selected observatories across Europe and the Red Sea. The raw sequence data are released in the European Nucleotide Archive (ENA) (Yuan et al. 2024) with metadata associated with the sampling event, sample preparation and sequencing procedures and a diverse set of measured environmental parameters available in the associated BioSamples (Courtot et al. 2021), such as temperature, salinity and nutrient concentrations.

This dataset contributes to the ongoing efforts of the Ocean Biodiversity Information System (OBIS), which aims at filling the gaps in our current knowledge on biodiversity of the world's oceans. Processed data will be published in OBIS and in the Global Biodiversity Information Facility (GBIF), using the DNA extension of the Darwin Core format. In addition, processed data, once available in OBIS and GBIF, will be incorporated in the European Marine Observation and Data Network (EMODnet) and the European Digital Twin of the Ocean (European DTO) initiatives.

## Methods

### Sampling

Sample collection was conducted under the standard operating procedures of the EMO BON Handbook (Santi et al. 2021). Water samples were collected and processed according to the "Water Column Standard Operating Procedures 1 ‒ WaSOP 1 (basic)": subsurface seawater was collected from the water column sampling site of each observatory, pre-filtered (> 200 μm) and concentrated by sequential filtration on polycarbonate (PC) membrane filters of 142 mm in diameter and pore sizes 3 µm and 0.2 µm. This resulted in two diﬀerent plankton size fractions: 3-200 μm and 0.2-3 μm. After filtration, each filter membrane was cut into two pieces by a sterile scalpel and each filter piece was considered to represent one replicate. In total, four replicates were collected from each sampling, since two separate sequential filtrations were conducted at each sampling site. Subsequenctly, membranes (replicates 1 and 2) were placed in individual containers with the DNA/RNA shield preservative (Zymo Research), flash frozen in liquid nitrogen and stored at -80^o^C until shipment to the sequencing facility. Filter membranes that were collected for biobanking (replicates 3 and 4) were preserved in cryotubes without the addition of DNA/RNA Shield and stored at -80^o^C.

Sediment samples were collected and processed, based on all the three proposed protocols. Briefly, observatories NRMCB and RiaFormosa used the 'Soft Substrate Standard Operating Procedures 1 – SoSOP 1 (intertidal sediments)", while OOB and ROSKOGO used the "Soft substrate Standard Operating Procedures 2 – SoSOP 2 (coastal sediments by diving)" and BPNS used "Soft substrate Standard Operating Procedures 3 ‒ SoSOP 3 (coastal sediments by research vessel)". Regardless of the choice of protocol, the steps regarding collection of sediment for microbial community assessment include the use of sediment cores and the subsequent slicing of the top 5 cm layer. As for the water samples, four replicates are collected for the sediment sampling; DNA/RNA shield was added in two of the replicates, which were the ones shipped for sequencing.

#### Geographic range

The dataset's geographical range includes 14 locations (13 observatories) across eight ecoregions, based on the Marine Ecoregions of the World (MEOW), proposed by Spalding et al. (2007) (Table 1; Fig. 1); details are also provided regarding the locality of the observatories, from the broader (ocean/sea) to the regional and the local scale.

#### DNA extraction, library preparation and sequencing

DNA extraction was performed at Genoscope, which was the chosen centralised facility to minimise biases and follow the same standardised procedures. For DNA extraction of the water column filter samples, the same protocol as described by Alberti et al. (2017) was used. The procedure consisted of a first step of cell disruption by cryogenic grinding of membrane filters followed by chemical lysis and then nucleic acid purification using NucleoSpin RNA kits, combined with the NucleoSpin RNA/DNA buffer set (Macherey-Nagel, Düren, Germany). For sediment samples, DNA extraction was performed using the commercially available DNeasy PowerSoil Pro Kit (Qiagen) with slight modifications.

Sequencing was also performed at Genoscope. Metagenome libraries were constructed according to the available DNA: 10 to 100 ng of genomic DNA were sonicated to obtain fragments of around 350 bp, using the Covaris E220 instrument (Covaris, Woburn, MA, USA). Fragments were repaired, 3'-adenylated and NEXTflex PCR freebarcodes adapters (Bioo Scientific, Austin, TX, USA) were added using the NEBNext® Ultra II DNALibrary prep kit for Illumina (New England Biolabs, Ipswich, MA, USA). Ligation products were purified by AMPure XP beads 0:8 volume (Beckmann Coulter, Brea, CA, USA). DNA fragments (> 200 bp) were amplified by PCR (2 PCR reactions, 14 cycles) using Illumina adapter-specific primers and NEBNext® Ultra II Q5 Master Mix (NEB). All libraries were subjected to size profile analysis conducted by Agilent 2100 Bioanalyzer (Agilent Technologies, Santa Clara, CA, USA) and to qPCR quantification using the KAPA Library Quantification Kit for Illumina Libraries (KapaBiosystems, Wilmington, MA, USA). All metagenomic libraries validated by the quality-control were sequenced using 151-bp pairwise read chemistry on an Illumina NovaSeq6000 sequencer, using S4 Flowcells (Illumina, San Diego, CA, USA). A minimum of 40,000 million useful paired-end reads were obtained per sample. Short Illumina reads were bioinformatically post-processed *sensu* Alberti et al. (2017) to filter out low-quality data. First, low-quality nucleotides (Q < 20) were discarded from both read ends. Then the remaining Illumina sequencing adapters and primer sequences were removed and only reads ≥ 30 nucleotides were retained. These filtering steps were done using in-house-designed software, based on the FastX package (Engelen and Aury 2016). Finally, read pairs mapping to the phage phiX genome were identifed and discarded using SOAP aligner (Li et al. (2008), default parameters) and the Enterobacteria phage PhiX174 reference sequence (GenBank: NC_001422.1).

### Biodiversity scope

#### Target

The target of the dataset was to assess prokaryotic and eukaryotic diversity associated with the collected samples.

#### Taxonomic range

Archaea, Bacteria, Eukaryota

## Data Resources

Details for the samples can be found in Suppl. material 1 and their basic metadata can be found in Suppl. material 2. All the raw sequence files of this study were submitted to ENA (Yuan et al. 2024) with the umbrella study accession number PRJEB51688. The accession numbers of the component projects under the umbrella study are PRJEB51662, PRJEB51661, PRJEB51660, PRJEB51659, PRJEB51658, PRJEB51665, PRJEB51664, PRJEB51656, PRJEB51655, PRJEB51654, PRJEB51653, PRJEB51652 and PRJEB50566. All sampling event and environmental data, linked to the respective accession numbers, are also available to browse and download from EMO BON’s data landing page.

### Resource 1

Download URL: ftp.sra.ebi.ac.uk/vol1/fastq/ERR139/037/ERR13930537/ERR13930537_1.fastq.gz

Resource identifier: ERR13930537

Data format : FASTQ

Download URL: ftp.sra.ebi.ac.uk/vol1/fastq/ERR139/037/ERR13930537/ERR13930537_2.fastq.gz

### Resource 2

Download URL: ftp.sra.ebi.ac.uk/vol1/fastq/ERR139/066/ERR13954066/ERR13954066_1.fastq.gz

Resource identifier: ERR13954066

Data format : FASTQ

Download URL: ftp.sra.ebi.ac.uk/vol1/fastq/ERR139/066/ERR13954066/ERR13954066_2.fastq.gz

### Resource 3

Download URL: ftp.sra.ebi.ac.uk/vol1/fastq/ERR139/067/ERR13954067/ERR13954067_1.fastq.gz

Resource identifier: ERR13954067

Data format : FASTQ

Download URL: ftp.sra.ebi.ac.uk/vol1/fastq/ERR139/067/ERR13954067/ERR13954067_2.fastq.gz

### Resource 4

Download URL: ftp.sra.ebi.ac.uk/vol1/fastq/ERR139/068/ERR13954068/ERR13954068_1.fastq.gz

Resource identifier: ERR13954068

Data format : FASTQ

Download URL: ftp.sra.ebi.ac.uk/vol1/fastq/ERR139/068/ERR13954068/ERR13954068_2.fastq.gz

### Resource 5

Download URL: ftp.sra.ebi.ac.uk/vol1/fastq/ERR139/069/ERR13954069/ERR13954069_1.fastq.gz

Resource identifier: ERR13954069

Data format : FASTQ

Download URL: ftp.sra.ebi.ac.uk/vol1/fastq/ERR139/069/ERR13954069/ERR13954069_2.fastq.gz

### Resource 6

Download URL: ftp.sra.ebi.ac.uk/vol1/fastq/ERR139/054/ERR13954254/ERR13954254_1.fastq.gz

Resource identifier: ERR13954254

Data format : FASTQ

Download URL: ftp.sra.ebi.ac.uk/vol1/fastq/ERR139/054/ERR13954254/ERR13954254_2.fastq.gz

### Resource 7

Download URL: ftp.sra.ebi.ac.uk/vol1/fastq/ERR139/055/ERR13954255/ERR13954255_1.fastq.gz

Resource identifier: ERR13954255

Data format : FASTQ

Download URL: ftp.sra.ebi.ac.uk/vol1/fastq/ERR139/055/ERR13954255/ERR13954255_2.fastq.gz

### Resource 8

Download URL: ftp.sra.ebi.ac.uk/vol1/fastq/ERR139/056/ERR13954256/ERR13954256_1.fastq.gz

Resource identifier: ERR13954256

Data format : FASTQ

Download URL: ftp.sra.ebi.ac.uk/vol1/fastq/ERR139/056/ERR13954256/ERR13954256_2.fastq.gz

### Resource 9

Download URL: ftp.sra.ebi.ac.uk/vol1/fastq/ERR139/057/ERR13954257/ERR13954257_1.fastq.gz

Resource identifier: ERR13954257

Data format : FASTQ

Download URL: ftp.sra.ebi.ac.uk/vol1/fastq/ERR139/057/ERR13954257/ERR13954257_2.fastq.gz

### Resource 10

Download URL: ftp.sra.ebi.ac.uk/vol1/fastq/ERR139/058/ERR13954258/ERR13954258_1.fastq.gz

Resource identifier: ERR13954258

Data format : FASTQ

Download URL: ftp.sra.ebi.ac.uk/vol1/fastq/ERR139/058/ERR13954258/ERR13954258_2.fastq.gz

### Resource 11

Download URL: ftp.sra.ebi.ac.uk/vol1/fastq/ERR139/059/ERR13954259/ERR13954259_1.fastq.gz

Resource identifier: ERR13954259

Data format : FASTQ

Download URL: ftp.sra.ebi.ac.uk/vol1/fastq/ERR139/059/ERR13954259/ERR13954259_2.fastq.gz

### Resource 12

Download URL: ftp.sra.ebi.ac.uk/vol1/fastq/ERR139/060/ERR13954260/ERR13954260_1.fastq.gz

Resource identifier: ERR13954260

Data format : FASTQ

Download URL: ftp.sra.ebi.ac.uk/vol1/fastq/ERR139/060/ERR13954260/ERR13954260_2.fastq.gz

### Resource 13

Download URL: ftp.sra.ebi.ac.uk/vol1/fastq/ERR139/064/ERR13954264/ERR13954264_1.fastq.gz

Resource identifier: ERR13954264

Data format : FASTQ

Download URL: ftp.sra.ebi.ac.uk/vol1/fastq/ERR139/064/ERR13954264/ERR13954264_2.fastq.gz

### Resource 14

Download URL: ftp.sra.ebi.ac.uk/vol1/fastq/ERR139/065/ERR13954265/ERR13954265_1.fastq.gz

Resource identifier: ERR13954265

Data format : FASTQ

Download URL: ftp.sra.ebi.ac.uk/vol1/fastq/ERR139/065/ERR13954265/ERR13954265_2.fastq.gz

### Resource 15

Download URL: ftp.sra.ebi.ac.uk/vol1/fastq/ERR139/066/ERR13954266/ERR13954266_1.fastq.gz

Resource identifier: ERR13954266

Data format : FASTQ

Download URL: ftp.sra.ebi.ac.uk/vol1/fastq/ERR139/066/ERR13954266/ERR13954266_2.fastq.gz

### Resource 16

Download URL: ftp.sra.ebi.ac.uk/vol1/fastq/ERR139/067/ERR13954267/ERR13954267_1.fastq.gz

Resource identifier: ERR13954267

Data format : FASTQ

Download URL: ftp.sra.ebi.ac.uk/vol1/fastq/ERR139/067/ERR13954267/ERR13954267_2.fastq.gz

### Resource 17

Download URL: ftp.sra.ebi.ac.uk/vol1/fastq/ERR139/068/ERR13954268/ERR13954268_1.fastq.gz

Resource identifier: ERR13954268

Data format : FASTQ

Download URL: ftp.sra.ebi.ac.uk/vol1/fastq/ERR139/068/ERR13954268/ERR13954268_2.fastq.gz

### Resource 18

Download URL: ftp.sra.ebi.ac.uk/vol1/fastq/ERR139/069/ERR13954269/ERR13954269_1.fastq.gz

Resource identifier: ERR13954269

Data format : FASTQ

Download URL: ftp.sra.ebi.ac.uk/vol1/fastq/ERR139/069/ERR13954269/ERR13954269_2.fastq.gz

### Resource 19

Download URL: ftp.sra.ebi.ac.uk/vol1/fastq/ERR139/070/ERR13954270/ERR13954270_1.fastq.gz

Resource identifier: ERR13954270

Data format : FASTQ

Download URL: ftp.sra.ebi.ac.uk/vol1/fastq/ERR139/070/ERR13954270/ERR13954270_2.fastq.gz

### Resource 20

Download URL: ftp.sra.ebi.ac.uk/vol1/fastq/ERR139/071/ERR13954271/ERR13954271_1.fastq.gz

Resource identifier: ERR13954271

Data format : FASTQ

Download URL: ftp.sra.ebi.ac.uk/vol1/fastq/ERR139/071/ERR13954271/ERR13954271_2.fastq.gz

### Resource 21

Download URL: ftp.sra.ebi.ac.uk/vol1/fastq/ERR139/073/ERR13954273/ERR13954273_1.fastq.gz

Resource identifier: ERR13954273

Data format : FASTQ

Download URL: ftp.sra.ebi.ac.uk/vol1/fastq/ERR139/073/ERR13954273/ERR13954273_2.fastq.gz

### Resource 22

Download URL: ftp.sra.ebi.ac.uk/vol1/fastq/ERR139/074/ERR13954274/ERR13954274_1.fastq.gz

Resource identifier: ERR13954274

Data format : FASTQ

Download URL: ftp.sra.ebi.ac.uk/vol1/fastq/ERR139/074/ERR13954274/ERR13954274_2.fastq.gz

### Resource 23

Download URL: ftp.sra.ebi.ac.uk/vol1/fastq/ERR139/075/ERR13954275/ERR13954275_1.fastq.gz

Resource identifier: ERR13954275

Data format : FASTQ

Download URL: ftp.sra.ebi.ac.uk/vol1/fastq/ERR139/075/ERR13954275/ERR13954275_2.fastq.gz

### Resource 24

Download URL: ftp.sra.ebi.ac.uk/vol1/fastq/ERR139/076/ERR13954276/ERR13954276_1.fastq.gz

Resource identifier: ERR13954276

Data format : FASTQ

Download URL: ftp.sra.ebi.ac.uk/vol1/fastq/ERR139/076/ERR13954276/ERR13954276_2.fastq.gz

### Resource 25

Download URL: ftp.sra.ebi.ac.uk/vol1/fastq/ERR139/079/ERR13954279/ERR13954279_1.fastq.gz

Resource identifier: ERR13954279

Data format : FASTQ

Download URL: ftp.sra.ebi.ac.uk/vol1/fastq/ERR139/079/ERR13954279/ERR13954279_2.fastq.gz

### Resource 26

Download URL: ftp.sra.ebi.ac.uk/vol1/fastq/ERR139/080/ERR13954280/ERR13954280_1.fastq.gz

Resource identifier: ERR13954280

Data format : FASTQ

Download URL: ftp.sra.ebi.ac.uk/vol1/fastq/ERR139/080/ERR13954280/ERR13954280_2.fastq.gz

### Resource 27

Download URL: ftp.sra.ebi.ac.uk/vol1/fastq/ERR139/081/ERR13954281/ERR13954281_1.fastq.gz

Resource identifier: ERR13954281

Data format : FASTQ

Download URL: ftp.sra.ebi.ac.uk/vol1/fastq/ERR139/081/ERR13954281/ERR13954281_2.fastq.gz

### Resource 28

Download URL: ftp.sra.ebi.ac.uk/vol1/fastq/ERR139/082/ERR13954282/ERR13954282_1.fastq.gz

Resource identifier: ERR13954282

Data format : FASTQ

Download URL: ftp.sra.ebi.ac.uk/vol1/fastq/ERR139/082/ERR13954282/ERR13954282_2.fastq.gz

### Resource 29

Download URL: ftp.sra.ebi.ac.uk/vol1/fastq/ERR139/083/ERR13954283/ERR13954283_1.fastq.gz

Resource identifier: ERR13954283

Data format : FASTQ

Download URL: ftp.sra.ebi.ac.uk/vol1/fastq/ERR139/083/ERR13954283/ERR13954283_2.fastq.gz

### Resource 30

Download URL: ftp.sra.ebi.ac.uk/vol1/fastq/ERR139/038/ERR13954638/ERR13954638_1.fastq.gz

Resource identifier: ERR13954638

Data format : FASTQ

Download URL: ftp.sra.ebi.ac.uk/vol1/fastq/ERR139/038/ERR13954638/ERR13954638_2.fastq.gz

### Resource 31

Download URL: ftp.sra.ebi.ac.uk/vol1/fastq/ERR139/039/ERR13954639/ERR13954639_1.fastq.gz

Resource identifier: ERR13954639

Data format : FASTQ

Download URL: ftp.sra.ebi.ac.uk/vol1/fastq/ERR139/039/ERR13954639/ERR13954639_2.fastq.gz

### Resource 32

Download URL: ftp.sra.ebi.ac.uk/vol1/fastq/ERR139/040/ERR13954640/ERR13954640_1.fastq.gz

Resource identifier: ERR13954640

Data format : FASTQ

Download URL: ftp.sra.ebi.ac.uk/vol1/fastq/ERR139/040/ERR13954640/ERR13954640_2.fastq.gz

### Resource 33

Download URL: ftp.sra.ebi.ac.uk/vol1/fastq/ERR139/041/ERR13954641/ERR13954641_1.fastq.gz

Resource identifier: ERR13954641

Data format : FASTQ

Download URL: ftp.sra.ebi.ac.uk/vol1/fastq/ERR139/041/ERR13954641/ERR13954641_2.fastq.gz

### Resource 34

Download URL: ftp.sra.ebi.ac.uk/vol1/fastq/ERR139/042/ERR13954642/ERR13954642_1.fastq.gz

Resource identifier: ERR13954642

Data format : FASTQ

Download URL: ftp.sra.ebi.ac.uk/vol1/fastq/ERR139/042/ERR13954642/ERR13954642_2.fastq.gz

### Resource 35

Download URL: ftp.sra.ebi.ac.uk/vol1/fastq/ERR139/043/ERR13954643/ERR13954643_1.fastq.gz

Resource identifier: ERR13954643

Data format : FASTQ

Download URL: ftp.sra.ebi.ac.uk/vol1/fastq/ERR139/043/ERR13954643/ERR13954643_2.fastq.gz

### Resource 36

Download URL: ftp.sra.ebi.ac.uk/vol1/fastq/ERR139/028/ERR13954728/ERR13954728_1.fastq.gz

Resource identifier: ERR13954728

Data format : FASTQ

Download URL: ftp.sra.ebi.ac.uk/vol1/fastq/ERR139/028/ERR13954728/ERR13954728_2.fastq.gz

### Resource 37

Download URL: ftp.sra.ebi.ac.uk/vol1/fastq/ERR139/029/ERR13954729/ERR13954729_1.fastq.gz

Resource identifier: ERR13954729

Data format : FASTQ

Download URL: ftp.sra.ebi.ac.uk/vol1/fastq/ERR139/029/ERR13954729/ERR13954729_2.fastq.gz

### Resource 38

Download URL: ftp.sra.ebi.ac.uk/vol1/fastq/ERR139/030/ERR13954730/ERR13954730_1.fastq.gz

Resource identifier: ERR13954730

Data format : FASTQ

Download URL: ftp.sra.ebi.ac.uk/vol1/fastq/ERR139/030/ERR13954730/ERR13954730_2.fastq.gz

### Resource 39

Download URL: ftp.sra.ebi.ac.uk/vol1/fastq/ERR139/031/ERR13954731/ERR13954731_1.fastq.gz

Resource identifier: ERR13954731

Data format : FASTQ

Download URL: ftp.sra.ebi.ac.uk/vol1/fastq/ERR139/031/ERR13954731/ERR13954731_2.fastq.gz

### Resource 40

Download URL: ftp.sra.ebi.ac.uk/vol1/fastq/ERR139/032/ERR13954732/ERR13954732_1.fastq.gz

Resource identifier: ERR13954732

Data format : FASTQ

Download URL: ftp.sra.ebi.ac.uk/vol1/fastq/ERR139/032/ERR13954732/ERR13954732_2.fastq.gz

### Resource 41

Download URL: ftp.sra.ebi.ac.uk/vol1/fastq/ERR139/033/ERR13954733/ERR13954733_1.fastq.gz

Resource identifier: ERR13954733

Data format : FASTQ

Download URL: ftp.sra.ebi.ac.uk/vol1/fastq/ERR139/033/ERR13954733/ERR13954733_2.fastq.gz

### Resource 42

Download URL: ftp.sra.ebi.ac.uk/vol1/fastq/ERR139/034/ERR13954734/ERR13954734_1.fastq.gz

Resource identifier: ERR13954734

Data format : FASTQ

Download URL: ftp.sra.ebi.ac.uk/vol1/fastq/ERR139/034/ERR13954734/ERR13954734_2.fastq.gz

### Resource 43

Download URL: ftp.sra.ebi.ac.uk/vol1/fastq/ERR139/035/ERR13954735/ERR13954735_1.fastq.gz

Resource identifier: ERR13954735

Data format : FASTQ

Download URL: ftp.sra.ebi.ac.uk/vol1/fastq/ERR139/035/ERR13954735/ERR13954735_2.fastq.gz

### Resource 44

Download URL: ftp.sra.ebi.ac.uk/vol1/fastq/ERR139/036/ERR13954736/ERR13954736_1.fastq.gz

Resource identifier: ERR13954736

Data format : FASTQ

Download URL: ftp.sra.ebi.ac.uk/vol1/fastq/ERR139/036/ERR13954736/ERR13954736_2.fastq.gz

### Resource 45

Download URL: ftp.sra.ebi.ac.uk/vol1/fastq/ERR139/037/ERR13954737/ERR13954737_1.fastq.gz

Resource identifier: ERR13954737

Data format : FASTQ

Download URL: ftp.sra.ebi.ac.uk/vol1/fastq/ERR139/037/ERR13954737/ERR13954737_2.fastq.gz

### Resource 46

Download URL: ftp.sra.ebi.ac.uk/vol1/fastq/ERR139/038/ERR13954738/ERR13954738_1.fastq.gz

Resource identifier: ERR13954738

Data format : FASTQ

Download URL: ftp.sra.ebi.ac.uk/vol1/fastq/ERR139/038/ERR13954738/ERR13954738_2.fastq.gz

### Resource 47

Download URL: ftp.sra.ebi.ac.uk/vol1/fastq/ERR139/039/ERR13954739/ERR13954739_1.fastq.gz

Resource identifier: ERR13954739

Data format : FASTQ

Download URL: ftp.sra.ebi.ac.uk/vol1/fastq/ERR139/039/ERR13954739/ERR13954739_2.fastq.gz

### Resource 48

Download URL: ftp.sra.ebi.ac.uk/vol1/fastq/ERR139/040/ERR13954740/ERR13954740_1.fastq.gz

Resource identifier: ERR13954740

Data format : FASTQ

Download URL: ftp.sra.ebi.ac.uk/vol1/fastq/ERR139/040/ERR13954740/ERR13954740_2.fastq.gz

### Resource 49

Download URL: ftp.sra.ebi.ac.uk/vol1/fastq/ERR139/013/ERR13954813/ERR13954813_1.fastq.gz

Resource identifier: ERR13954813

Data format : FASTQ

Download URL: ftp.sra.ebi.ac.uk/vol1/fastq/ERR139/013/ERR13954813/ERR13954813_2.fastq.gz

### Resource 50

Download URL: ftp.sra.ebi.ac.uk/vol1/fastq/ERR139/014/ERR13954814/ERR13954814_1.fastq.gz

Resource identifier: ERR13954814

Data format : FASTQ

Download URL: ftp.sra.ebi.ac.uk/vol1/fastq/ERR139/014/ERR13954814/ERR13954814_2.fastq.gz

### Resource 51

Download URL: ftp.sra.ebi.ac.uk/vol1/fastq/ERR139/015/ERR13954815/ERR13954815_1.fastq.gz

Resource identifier: ERR13954815

Data format : FASTQ

Download URL: ftp.sra.ebi.ac.uk/vol1/fastq/ERR139/015/ERR13954815/ERR13954815_2.fastq.gz

### Resource 52

Download URL: ftp.sra.ebi.ac.uk/vol1/fastq/ERR139/016/ERR13954816/ERR13954816_1.fastq.gz

Resource identifier: ERR13954816

Data format : FASTQ

Download URL: ftp.sra.ebi.ac.uk/vol1/fastq/ERR139/016/ERR13954816/ERR13954816_2.fastq.gz

### Resource 53

Download URL: ftp.sra.ebi.ac.uk/vol1/fastq/ERR139/017/ERR13954817/ERR13954817_1.fastq.gz

Resource identifier: ERR13954817

Data format : FASTQ

Download URL: ftp.sra.ebi.ac.uk/vol1/fastq/ERR139/017/ERR13954817/ERR13954817_2.fastq.gz

### Resource 54

Download URL: ftp.sra.ebi.ac.uk/vol1/fastq/ERR139/018/ERR13954818/ERR13954818_1.fastq.gz

Resource identifier: ERR13954818

Data format : FASTQ

Download URL: ftp.sra.ebi.ac.uk/vol1/fastq/ERR139/018/ERR13954818/ERR13954818_2.fastq.gz

### Resource 55

Download URL: ftp.sra.ebi.ac.uk/vol1/fastq/ERR139/019/ERR13954819/ERR13954819_1.fastq.gz

Resource identifier: ERR13954819

Data format : FASTQ

Download URL: ftp.sra.ebi.ac.uk/vol1/fastq/ERR139/019/ERR13954819/ERR13954819_2.fastq.gz

### Resource 56

Download URL: ftp.sra.ebi.ac.uk/vol1/fastq/ERR139/020/ERR13954820/ERR13954820_1.fastq.gz

Resource identifier: ERR13954820

Data format : FASTQ

Download URL: ftp.sra.ebi.ac.uk/vol1/fastq/ERR139/020/ERR13954820/ERR13954820_2.fastq.gz

### Resource 57

Download URL: ftp.sra.ebi.ac.uk/vol1/fastq/ERR139/052/ERR13954852/ERR13954852_1.fastq.gz

Resource identifier: ERR13954852

Data format : FASTQ

Download URL: ftp.sra.ebi.ac.uk/vol1/fastq/ERR139/052/ERR13954852/ERR13954852_2.fastq.gz

### Resource 58

Download URL: ftp.sra.ebi.ac.uk/vol1/fastq/ERR139/053/ERR13954853/ERR13954853_1.fastq.gz

Resource identifier: ERR13954853

Data format : FASTQ

Download URL: ftp.sra.ebi.ac.uk/vol1/fastq/ERR139/053/ERR13954853/ERR13954853_2.fastq.gz

### Resource 59

Download URL: ftp.sra.ebi.ac.uk/vol1/fastq/ERR139/054/ERR13954854/ERR13954854_1.fastq.gz

Resource identifier: ERR13954854

Data format : FASTQ

Download URL: ftp.sra.ebi.ac.uk/vol1/fastq/ERR139/054/ERR13954854/ERR13954854_2.fastq.gz

### Resource 60

Download URL: ftp.sra.ebi.ac.uk/vol1/fastq/ERR139/055/ERR13954855/ERR13954855_1.fastq.gz

Resource identifier: ERR13954855

Data format : FASTQ

Download URL: ftp.sra.ebi.ac.uk/vol1/fastq/ERR139/055/ERR13954855/ERR13954855_2.fastq.gz

### Resource 61

Download URL: ftp.sra.ebi.ac.uk/vol1/fastq/ERR139/056/ERR13954856/ERR13954856_1.fastq.gz

Resource identifier: ERR13954856

Data format : FASTQ

Download URL: ftp.sra.ebi.ac.uk/vol1/fastq/ERR139/056/ERR13954856/ERR13954856_2.fastq.gz

### Resource 62

Download URL: ftp.sra.ebi.ac.uk/vol1/fastq/ERR139/057/ERR13954857/ERR13954857_1.fastq.gz

Resource identifier: ERR13954857

Data format : FASTQ

Download URL: ftp.sra.ebi.ac.uk/vol1/fastq/ERR139/057/ERR13954857/ERR13954857_2.fastq.gz

### Resource 63

Download URL: ftp.sra.ebi.ac.uk/vol1/fastq/ERR139/058/ERR13954858/ERR13954858_1.fastq.gz

Resource identifier: ERR13954858

Data format : FASTQ

Download URL: ftp.sra.ebi.ac.uk/vol1/fastq/ERR139/058/ERR13954858/ERR13954858_2.fastq.gz

### Resource 64

Download URL: ftp.sra.ebi.ac.uk/vol1/fastq/ERR139/059/ERR13954859/ERR13954859_1.fastq.gz

Resource identifier: ERR13954859

Data format : FASTQ

Download URL: ftp.sra.ebi.ac.uk/vol1/fastq/ERR139/059/ERR13954859/ERR13954859_2.fastq.gz

### Resource 65

Download URL: ftp.sra.ebi.ac.uk/vol1/fastq/ERR139/060/ERR13954860/ERR13954860_1.fastq.gz

Resource identifier: ERR13954860

Data format : FASTQ

Download URL: ftp.sra.ebi.ac.uk/vol1/fastq/ERR139/060/ERR13954860/ERR13954860_2.fastq.gz

### Resource 66

Download URL: ftp.sra.ebi.ac.uk/vol1/fastq/ERR139/061/ERR13954861/ERR13954861_1.fastq.gz

Resource identifier: ERR13954861

Data format : FASTQ

Download URL: ftp.sra.ebi.ac.uk/vol1/fastq/ERR139/061/ERR13954861/ERR13954861_2.fastq.gz

### Resource 67

Download URL: ftp.sra.ebi.ac.uk/vol1/fastq/ERR139/062/ERR13954862/ERR13954862_1.fastq.gz

Resource identifier: ERR13954862

Data format : FASTQ

Download URL: ftp.sra.ebi.ac.uk/vol1/fastq/ERR139/062/ERR13954862/ERR13954862_2.fastq.gz

### Resource 68

Download URL: ftp.sra.ebi.ac.uk/vol1/fastq/ERR139/063/ERR13954863/ERR13954863_1.fastq.gz

Resource identifier: ERR13954863

Data format : FASTQ

Download URL: ftp.sra.ebi.ac.uk/vol1/fastq/ERR139/063/ERR13954863/ERR13954863_2.fastq.gz

### Resource 69

Download URL: ftp.sra.ebi.ac.uk/vol1/fastq/ERR139/064/ERR13954864/ERR13954864_1.fastq.gz

Resource identifier: ERR13954864

Data format : FASTQ

Download URL: ftp.sra.ebi.ac.uk/vol1/fastq/ERR139/064/ERR13954864/ERR13954864_2.fastq.gz

### Resource 70

Download URL: ftp.sra.ebi.ac.uk/vol1/fastq/ERR139/087/ERR13955087/ERR13955087_1.fastq.gz

Resource identifier: ERR13955087

Data format : FASTQ

Download URL: ftp.sra.ebi.ac.uk/vol1/fastq/ERR139/087/ERR13955087/ERR13955087_2.fastq.gz

### Resource 71

Download URL: ftp.sra.ebi.ac.uk/vol1/fastq/ERR139/088/ERR13955088/ERR13955088_1.fastq.gz

Resource identifier: ERR13955088

Data format : FASTQ

Download URL: ftp.sra.ebi.ac.uk/vol1/fastq/ERR139/088/ERR13955088/ERR13955088_2.fastq.gz

### Resource 72

Download URL: ftp.sra.ebi.ac.uk/vol1/fastq/ERR139/089/ERR13955089/ERR13955089_1.fastq.gz

Resource identifier: ERR13955089

Data format : FASTQ

Download URL: ftp.sra.ebi.ac.uk/vol1/fastq/ERR139/089/ERR13955089/ERR13955089_2.fastq.gz

### Resource 73

Download URL: ftp.sra.ebi.ac.uk/vol1/fastq/ERR139/090/ERR13955090/ERR13955090_1.fastq.gz

Resource identifier: ERR13955090

Data format : FASTQ

Download URL: ftp.sra.ebi.ac.uk/vol1/fastq/ERR139/090/ERR13955090/ERR13955090_2.fastq.gz

### Resource 74

Download URL: ftp.sra.ebi.ac.uk/vol1/fastq/ERR139/091/ERR13955091/ERR13955091_1.fastq.gz

Resource identifier: ERR13955091

Data format : FASTQ

Download URL: ftp.sra.ebi.ac.uk/vol1/fastq/ERR139/091/ERR13955091/ERR13955091_2.fastq.gz

### Resource 75

Download URL: ftp.sra.ebi.ac.uk/vol1/fastq/ERR139/092/ERR13955092/ERR13955092_1.fastq.gz

Resource identifier: ERR13955092

Data format : FASTQ

Download URL: ftp.sra.ebi.ac.uk/vol1/fastq/ERR139/092/ERR13955092/ERR13955092_2.fastq.gz

### Resource 76

Download URL: ftp.sra.ebi.ac.uk/vol1/fastq/ERR139/093/ERR13955093/ERR13955093_1.fastq.gz

Resource identifier: ERR13955093

Data format : FASTQ

Download URL: ftp.sra.ebi.ac.uk/vol1/fastq/ERR139/093/ERR13955093/ERR13955093_2.fastq.gz

### Resource 77

Download URL: ftp.sra.ebi.ac.uk/vol1/fastq/ERR139/094/ERR13955094/ERR13955094_1.fastq.gz

Resource identifier: ERR13955094

Data format : FASTQ

Download URL: ftp.sra.ebi.ac.uk/vol1/fastq/ERR139/094/ERR13955094/ERR13955094_2.fastq.gz

### Resource 78

Download URL: ftp.sra.ebi.ac.uk/vol1/fastq/ERR139/095/ERR13955095/ERR13955095_1.fastq.gz

Resource identifier: ERR13955095

Data format : FASTQ

Download URL: ftp.sra.ebi.ac.uk/vol1/fastq/ERR139/095/ERR13955095/ERR13955095_2.fastq.gz

### Resource 79

Download URL: ftp.sra.ebi.ac.uk/vol1/fastq/ERR139/096/ERR13955096/ERR13955096_1.fastq.gz

Resource identifier: ERR13955096

Data format : FASTQ

Download URL: ftp.sra.ebi.ac.uk/vol1/fastq/ERR139/096/ERR13955096/ERR13955096_2.fastq.gz

### Resource 80

Download URL: ftp.sra.ebi.ac.uk/vol1/fastq/ERR139/097/ERR13955097/ERR13955097_1.fastq.gz

Resource identifier: ERR13955097

Data format : FASTQ

Download URL: ftp.sra.ebi.ac.uk/vol1/fastq/ERR139/097/ERR13955097/ERR13955097_2.fastq.gz

## Usage Rights

CC BY 4.0

Usage Rights

## Supplementary Material

87A5B609-47FE-53F9-A647-86032744143D 10.3897/BDJ.13.e143585.suppl1 Supplementary material 1 ENA sample, run, experiment and project accession numbers for the first release of the EMO BON shotgun metagenomics data from water and sediment samples Data type metadata File: oo_1178847.tsv https://binary.pensoft.net/file/1178847 Christina Pavloudi

16F035D2-8DAE-546D-BC25-474EF2E57FC6 10.3897/BDJ.13.e143585.suppl2 Supplementary material 2 BioSamples MIxS checklists Data type metadata File: oo_1178848.tsv https://binary.pensoft.net/file/1178848 Christina Pavloudi

## Figures and Tables

**Figure 1a.**
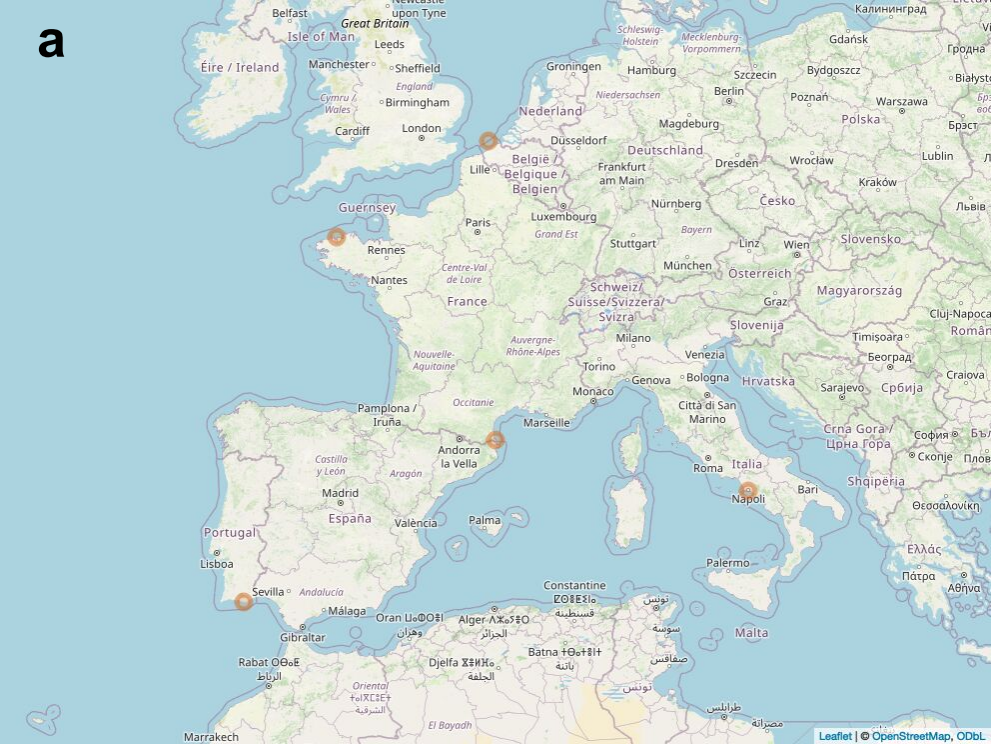
sediment samples;

**Figure 1b.**
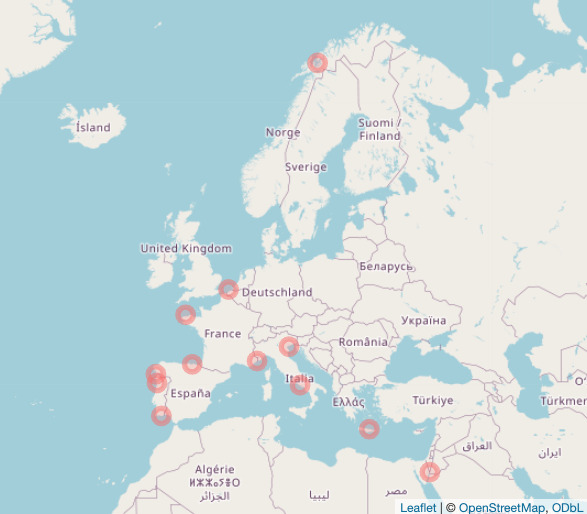
water samples.

**Table 1.** Locality and coordinates of the sampling stations.

**Observatory**	**Coordinates**	**Marine Ecoregion of the World (MEOW)**	**Ocean/Sea**	**Region**	**Location**	**Water sampling**	**Sediment sampling**	**Number of collected samples**	**Number of successfully sequenced samples**
AAOT	45.31417 N; 12.508333 E	Adriatic Sea	Mediterranean Sea - Eastern Basin	Adriatic Sea	Gulf of Venice	Yes		8	7
BPNS	51.43333 N; 2.808331 E	North Sea	North Atlantic Ocean	North Sea	Belgian part of the North Sea	Yes	Yes	12	11
EMT21	42.20194 N; -8.798500 E	South European Atlantic Shelf	Atlantic Ocean	North Atlantic Ocean	Vigo Seamount	Yes		4	4
ESC68N	68.92589 N; 17.125619 E	Northern Norway and Finnmark	Arctic Ocean	Norwegian Sea	Norwegian part of the Norwegian Sea	Yes		4	4
HCMR-1	35.34662 N; 25.278761 E	Aegean Sea	Mediterranean Sea - Eastern Basin	Aegean Sea	Crete Sea	Yes		12	5
IUIEilat	29.50000 N; 34.916667 E	Northern and Central Red Sea	Indian Ocean	Gulf of Eilat	Gulf of Eilat	Yes		4	4
NRMCB	40.80014 N; 14.250000 E	Western Mediterranean	Mediterranean Sea - Western Basin	Tyrrhenian Sea	Naples Gulf	Yes	Yes	18	13
OOB	42.48417 N; 3.135278 E	Western Mediterranean	Mediterranean Sea - Western Basin	Gulf of Lion	Bay of Banyuls-sur-Mer		Yes	3	1
OSD74	41.14653 N; -8.666639 E	South European Atlantic Shelf	Atlantic Ocean	North Atlantic Ocean	Porto Valley	Yes		3	3
PiEGetxo	43.33858 N; -3.014639 E	South European Atlantic Shelf	North Atlantic Ocean	Bay of Biscay	Abra de Bilbao	Yes		6	6
RFormosa	37.00564 N; -7.969250 E	South European Atlantic Shelf	Atlantic Ocean	North Atlantic Ocean	Ria Formosa	Yes	Yes	6	6
ROSKOGO	48.70833 N; -3.866000 E	Celtic Seas	North Atlantic Ocean	English Channel	French part of the English Channel		Yes	2	2
ROSKOGO	48.77167 N; -3.968333 E	Celtic Seas	North Atlantic Ocean	English Channel	French part of the English Channel	Yes		8	6
VB	43.68300 N; 7.317000 E	Western Mediterranean	Mediterranean Sea - Western Basin	Villefranche Bay	Villefranche Bay - Point B	Yes		8	8
